# Annual Research Review: The role of caregiver sensitivity in children's developmental outcomes – an umbrella review

**DOI:** 10.1111/jcpp.70087

**Published:** 2026-01-07

**Authors:** Marissa D. Nivison, Pasco Fearon, Jennifer M. Jenkins, Sheri Madigan

**Affiliations:** ^1^ Department of Psychology University of Calgary Calgary AB Canada; ^2^ Alberta Children's Hospital Research Institute Calgary AB Canada; ^3^ Centre for Child, Adolescent and Family Research University of Cambridge Cambridge UK; ^4^ Department of Applied Psychology and Human Development University of Toronto Toronto ON Canada

**Keywords:** Caregiver sensitivity, umbrella review, meta‐analysis

## Abstract

Caregiver sensitivity is the extent to which a caregiver notices a child's signal, interprets it correctly, and responds quickly and appropriately. Although originally introduced to developmental science as the key antecedent of attachment security, decades since its conception, hundreds of studies have been conducted examining the predictive significance of caregiver sensitivity to a broad range of developmental outcomes. The literature on caregiver sensitivity and related constructs (e.g., warmth, responsivity, negative parenting) has grown exponentially and is now the focus of several meta‐analyses. We conducted an umbrella review – a systematic review of reviews – to examine the extent to which caregiver sensitivity and related constructs are associated with child attachment, socioemotional, and cognitive outcomes. Searches in EMBASE, PsycINFO, and Medline and yielded 2,157 abstracts. Studies were included if they were a meta‐analysis of caregiver sensitivity or a related construct, focused on children's developmental outcomes, were available in English, French, or Spanish, and were published between 2010 and 2024. Conducted and reported in accordance with PRISMA guidelines, 17 meta‐analyses were identified. Using the *metaumbrella* package in *R*, we conducted quantitative analyses which demonstrated that caregiver sensitivity was moderately associated with attachment security (*r* = .25, *k* = 253, *n* = 37,444), cognition (*r* = .23, *k* = 44, *n* = 6,777), language skills (*r* = .26, *k* = 54, *n* = 11,136), and weakly associated with socioemotional problems (*r* = −.07, *k* = 135, *n* = 33,305). Narrative analysis of other meta‐analyses on caregiver warmth, responsivity, positive and negative parenting, and child outcomes also showed associations in the expected direction. Our findings demonstrate the critical importance of caregiver sensitivity on children's socioemotional and cognitive development, supporting caregiver sensitivity as an important target for early childhood prevention and intervention programs.

## Introduction

Caregiver sensitivity refers to a caregiver's ability to notice a child's signals, interpret the signals correctly, and respond quickly and appropriately. Originally introduced in developmental science as an antecedent of attachment security (Ainsworth, Blehar, Waters, & Wall, 1978), which has been strongly supported (Madigan et al., 2024), the construct has since been extensively studied for decades in the broader context of child development (Mesman & Emmen, 2016). Specifically, beyond its foundational link to attachment quality, caregiver sensitivity has been consistently associated with a wide range of developmental outcomes, including child temperament (Belsky, Hsieh, & Crnic, 1998), language skills (Madigan et al., 2019), cognition (Deneault et al., 2023; Rodrigues et al., 2021), academic achievement (Raby, Roisman, Fraley, & Simpson, 2015), physical health (Rhee, 2008), and mental health (Cooke et al., 2022; Rodrigues et al., 2021), cementing its role as a primary antecedent of children's developmental success.

The construct of caregiver sensitivity has been so influential in developmental science that it has been embedded in major international early child development policies, most notably the Nurturing Care Framework for Early Child Development hosted by the World Health Organization, the World Bank, and UNICEF, and in the Harvard Center for the Developing Child's concept of Serve and Return interactions. Accordingly, the promotion of sensitive caregiving interactions for young children and addressing the circumstances and risk factors that affect them has become widely accepted as a cornerstone of public health policy for young children, including policies relating to home care, parental mental health, and practice in early childhood care settings.

The goal of the present umbrella review, also known as a “review of reviews” (Ioannidis, 2009), is to systematically synthesize the meta‐analytic literature examining caregiver sensitivity in relation to specific aspects of child development, such as attachment security, language, cognition, and socioemotional development, within one comprehensive set of analyses. By integrating this previously fragmented body of meta‐analytic work we aim to clarify the strength of associations between caregiver sensitivity and child outcomes. More broadly, this review seeks to inform early child development policy and practice.

### Caregiver sensitivity: development and measurement

The concept of caregiver sensitivity was initially developed by Ainsworth, which she operationalized in an observational tool known as the *Sensitivity versus Insensitivity Scale* (Ainsworth, Bell, & Stayton, 1974). This observational scale was designed to evaluate sensitivity in the context of observed parent–child interactions for a child aged 12 months at home. At its heart, the scale captures the tendency a caregiver demonstrates in being aware of a child's subtle or not‐so‐subtle cues and communications, to interpret those cues and communications accurately, to respond to them in a timely and contingent manner, and to respond in a way that appears well suited to what the child appears to need, given their signals and the context.

Over time, the measurement of sensitivity has expanded across different developmental stages, with some studies assessing sensitivity as early as 1 month of age (Heinicke, Diskin, Ramsey‐Klee, & Oates, 1986), whereas others have extended measurements to interactions with children as old as 19 years (Green, 2018). Sensitivity has also been extended beyond its initial observational context and is now used in a variety of developmentally relevant contexts. For example, play or feeding sessions between parents and children are often used to assess caregiver sensitivity at 6 months of age, but as the child develops, semi‐structured tasks that are intentionally designed to be too difficult for the child to complete on their own are used (Nivison, Vandell, Booth‐LaForce, & Roisman, 2021).

As the research on sensitivity has expanded, so too has its operationalization and measurement. An extensive review conducted by Mesman and Emmen (2016) identified over 50 different observational instruments claiming to assess caregiver sensitivity. The authors identified eight instruments that contain key elements of Ainsworth's original coding system. These instruments include the CARE‐Index, the Coding Interactive Behavior Scale, Emotional Availability Scales, Erikson's Scales, Global Ratings of Mother‐Infant Interaction, Maternal Behavior Q‐Sort, the NICHD Study of Early Child Care and Youth Development Scales, and the Parent–Child Early Relational Assessment Scale (see Mesman & Emmen, 2016 for further details).

In 1997, De Wolff and van IJzendoorn conducted the first major effort to organize and synthesize the diverse operationalizations of caregiver sensitivity into meaningful groups. Using a card sorting task, they had experts sort 40 cards containing the names, definitions, and methods of measurement of constructs and asked them to group the cards into no more than 10 groups. Overall, the expert sorters established five groups of constructs captured by the diverse set of instruments: synchrony, mutuality, positive attitude, emotional support, and stimulation. Synchrony contained constructs such as coordinated social play and synchronous interactions. Mutuality contained constructs including harmony and positive mutuality. Positive attitude contained constructs such as warmth and positive interaction. Emotional support included constructs such as involvement and quality support. Finally, stimulation included constructs such as encouragement and effective stimulation. The results of De Wolff and van IJzendoorn's (1997) card sorting task demonstrated that the overall effect size between parenting and attachment quality was largely similar across Ainsworth's Sensitivity scale and these other related constructs. However, insights from De Wolff and van IJzendoorn's (1997) seminal paper have not been thoroughly followed up – namely that a wider range of parental behaviors may be linked to attachment security than those captured by Ainsworth's original work. The authors concluded that in the case of attachment quality, examining these other parenting constructs beyond sensitivity is important.

### Caregiver sensitivity: predictive significance

Caregiver sensitivity was originally developed to study the interactive antecedents of parent–child attachment security (Ainsworth et al., 1974). Decades later, caregiver sensitivity has remained one of the most thoroughly studied antecedents of both maternal (*r* = .26) and paternal attachment security (*r* = .21; Madigan et al., 2024). However, it was also quickly adopted as an important construct beyond attachment security. Individual meta‐analyses synthesizing hundreds of studies have demonstrated that maternal sensitivity is positively associated with children's cognitive development (*r* = .17; Deneault et al., 2023) and language skills (*r* = .27; Madigan et al., 2019) and negatively associated with socioemotional problems (internalizing symptoms, *r* = −.08; externalizing problems, *r* = −.14; Cooke et al., 2022). Similarly, paternal sensitivity plays a central role in children's developmental competencies, including their cognitive ability (*r* = .18), language skills (*r* = .21), executive functioning (*r* = .09), internalizing problems (*r* = −.02), externalizing problems (*r* = −.08), emotion regulation (*r* = .22), and attachment security (*r* = .25; Rodrigues et al., 2021). As evidence of the widespread impact of caregiver sensitivity on child outcomes continues to grow, it has been recognized as a key mechanism of change in attachment‐based interventions (Bakermans‐Kranenburg, van IJzendoorn, & Juffer, 2003) and public health campaigns (e.g., Center on the Developing Child at Harvard University, 2025).

### Caregiver sensitivity and child outcomes: theoretical underpinnings

Through repeated experiences of attachment‐related needs and communications being responded to sensitively and appropriately, a child is thought to develop a set of generalized cognitive‐affective representations that guide their expectations about caregiver availability and promote secure attachment behavior (Ainsworth et al., 1978). Several plausible mechanisms have been discussed in the literature as to how sensitivity influences other aspects of child development.

In terms of language and cognition, van IJzendoorn, Dijkstra, and Bus (1995) proposed four different theoretical explanations. First, a parent's sensitive responses to a child's cues in a learning or teaching context may provide optimal support or scaffolding for the child to understand and learn about the world around them. Second, sensitive caregivers may encourage exploration of the environment, which may promote learning. Third, sensitive parenting may teach children about reciprocal and positive interactions, which can be generalized to other relationships (such as with teachers), to promote learning. Finally, and closely relatedly, sensitive caregiving may foster cooperation and compliance, which children can internalize and apply to instances beyond the parent–child relationship (for a full review, see Deneault et al., 2023).

Other accounts have attempted to explicate the association between caregiver sensitivity and child language skills using similar lines of reasoning. Madigan, Plamondon, and Jenkins (2023), for example, postulated that by attending to and remaining responsive to a child's cues, a sensitive caregiver operates within the child's zone of proximal development by providing scaffolds to foster a child's ability to understand, use, and develop language, and in turn, these behaviors build the neural architecture for language (Romeo et al., 2018). Of course, in addition, the mechanisms noted by van IJzendoorn et al. (1995) in relation to cognition may also apply to varying extents to language learning (promoting exploration, promoting reciprocity, and promoting compliance).

Several theories have also sought to explain the association between caregiver sensitivity and child socioemotional functioning. According to emotion socialization theory (Eisenberg, Cumberland, & Spinrad, 1998) and social learning theory (Bandura, 1969; Grusec, 1994), children shape their own behaviors by observing their parents' actions and responses. For example, if a caregiver is dismissive, a child may learn to be withdrawn, potentially becoming uncomfortable with emotional expression over time, which may lead to difficulties with socioemotional regulation. Conversely, harsh and/or punitive responses to a child's behaviors can create escalating negative cycles of interaction, reinforcing the child's display of negative, angry, or defiant behaviors or distress, which in turn may elicit negative caregiving (Patterson, 1982).

Given that these parenting behaviors overlap with the (in)sensitivity construct, understanding sensitivity through the lens of these social learning mechanisms may provide insights into its role in shaping child outcomes. Furthermore, attachment theory postulates that continued exposure to insensitive caregiving may shape children's own internal working models of relationships – for example, parenting that is insensitive, withdrawn, or neglectful may lead a child to learn that their parent does not help in times of distress. This, in turn, may generalize to other relationships, which may lead to poorer socioemotional outcomes such as lower social competence, poor coping skills, and more symptoms of psychopathology (Cooke et al., 2022). Teasing apart these different mechanisms is a challenging and important task, and, as we note later, few studies have attempted it. Nevertheless, understanding the extent to which sensitivity has similar or different associations with a range of outcomes may provide important clues about the kinds of mechanisms through which it might be operating, and spur on future mechanistic research.

In summary, the predictive significance of sensitivity has been examined in various contexts and has been the subject of numerous meta‐analyses (e.g., Brumariu, Obsuth, & Lyons‐Ruth, 2013; Cooke et al., 2022; Deneault et al., 2023; Madigan et al., 2024). However, to date, each child outcome (i.e., attachment, language, cognition, behavioral problems) has largely been examined in isolation within individual meta‐analyses, resulting in a fragmented understanding of the overall impact of caregiver sensitivity on child development. Given the potential significance of caregiving for both practice and policy, a comprehensive quantitative synthesis of the meta‐analytic evidence is both timely and necessary.

### Umbrella review methodology

**Figure 1:**
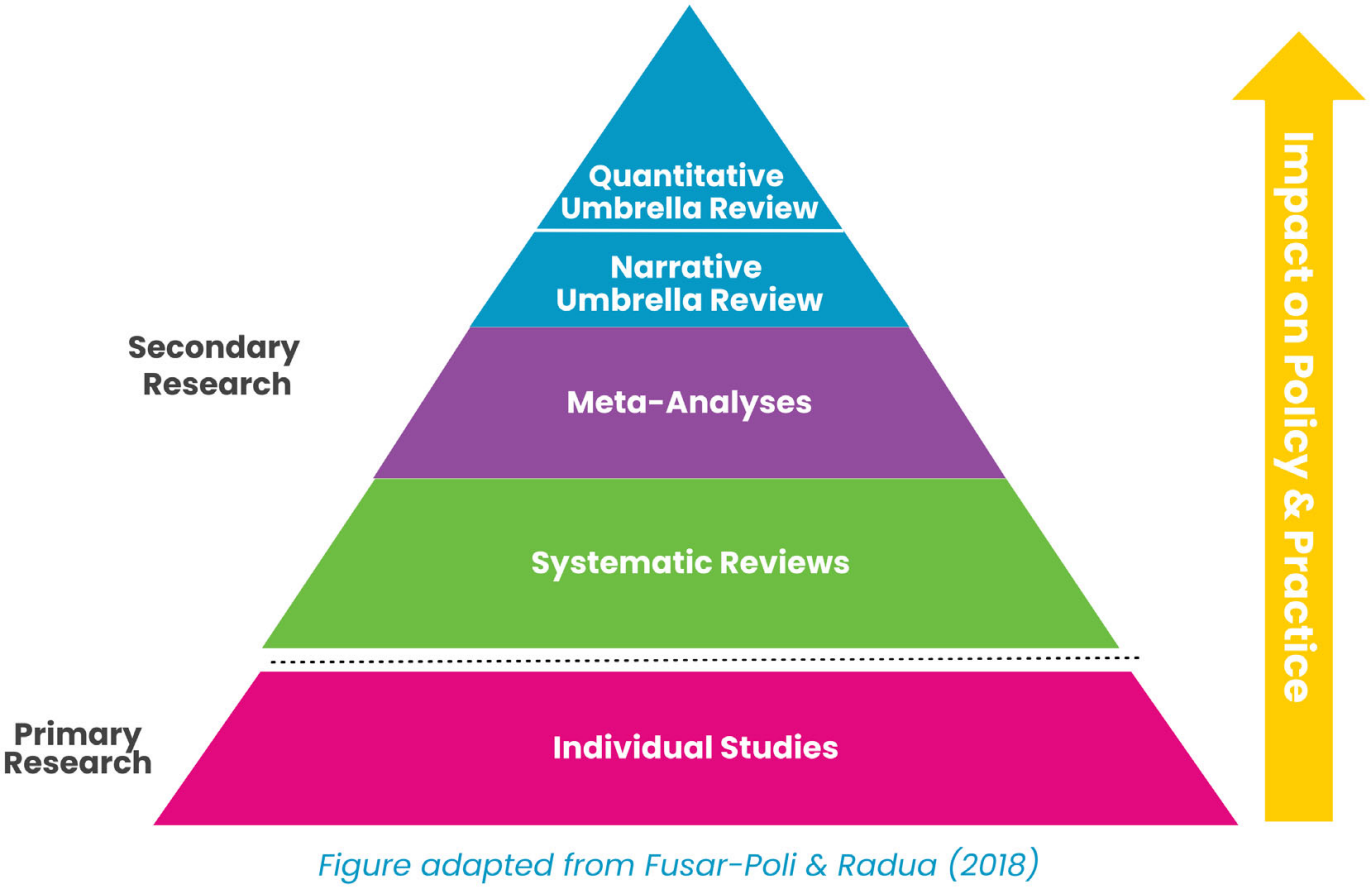
Figure is adapted from Fusar‐Poli and Radua (2018)

An innovative methodology from the biomedical literature, and newer to developmental science, is the umbrella review (see Ioannidis, 2009). An umbrella review is a systematic review of all meta‐analyses (and/or systematic reviews) on a single subject area. Historically, systematic reviews and meta‐analyses were the main way in which evidence was aggregated at a higher level from primary studies. However, with so many meta‐analyses conducted on one topic, the information became difficult to digest or appraise. Therefore, umbrella review methodologies have recently been developed to synthesize knowledge after the mass influx of meta‐analyses (or systematic reviews) have been published. They are the most succinct way to summarize the literature on a certain topic (see Figure 1) and can be particularly impactful in shaping policy and practice.

An umbrella review systematically collects meta‐analyses on a specific construct through structured database searches, enabling the generation of comparable estimates across studies (Fusar‐Poli & Radua, 2018). Traditionally, umbrella reviews provided simple descriptive results and graphs, but recent advances now enable more sophisticated statistical aggregation across meta‐analyses. Specifically, the *metaumbrella* package (Gosling, Solanes, Fusar‐Poli, & Radua, 2023) was developed in *R* to facilitate this type of advanced analysis.

In addition to estimating overall effect sizes, umbrella reviews now enable the examination of moderators that might influence the strength of associations. In the present review, we explored whether associations between caregiver sensitivity and child outcomes varied by key demographic or study design variables. These included child age and biological sex, parent gender, socioeconomic status (SES), and study design – factors commonly considered in prior meta‐analyses (e.g., Cooke et al., 2022; Deneault et al., 2023; Madigan et al., 2024). However, due to inconsistencies in how moderators were defined and coded across meta‐analyses (e.g., SES income versus education), we restricted our moderator analyses to those variables that were consistently defined and used across all the included meta‐analyses.

### The present umbrella review

The present umbrella review included meta‐analyses that examined the predictive significance of caregiver sensitivity, as well as broader constructs related to sensitivity as outlined by previous work (De Wolff & Van Ijzendoorn, 1997; Mesman & Emmen, 2016). Examples of related constructs included warmth, responsivity, and negative parenting. The present umbrella review had two main research questions:

1. To what extent is caregiver sensitivity meta‐analytically associated with, or predictive of, a broad array of developmental outcomes, such as language, cognition, socioemotional problems, and which factors moderate these associations?
2. How are related constructs of sensitivity meta‐analytically associated with developmental outcomes?

Aim 1 focused specifically on the construct of sensitivity using measures outlined by Mesman and Emmen (2016), which allowed us to conduct statistical analyses (i.e., a “mega‐analysis”) aggregating results from two or more meta‐analyses with similar outcomes and presenting the overall meta‐analytic effect across several child development domains. Given the wide variety of definitions in caregiving behaviors and the limited number of meta‐analyses on these constructs, only narrative results are presented for Aim 2.

## Methods

### Search strategy

A systematic search was conducted for meta‐analyses between January 1, 2010 and January 23, 2024. Dates were restricted to reduce the chance of overlapping meta‐analyses, consistent with prior umbrella reviews (e.g., Fusar‐Poli & Radua, 2018; van IJzendoorn, Bakermans‐Kranenburg, Coughlan, & Reijman, 2020). The following electronic databases were searched: EMBASE, PsycINFO, and Medline, and 1,555 nonduplicate abstracts were identified (see Appendix S1 for full search strategy).

### Inclusion and exclusion criteria

Studies were included in the umbrella review if they met the following criteria: a meta‐analytic study of caregiver sensitivity, or a related construct (e.g., warmth, responsiveness, negative parenting, synchrony), focused on the association of caregiver sensitivity for children's development, and available in English, French, or Spanish (the languages spoken by our team). Systematic reviews without a formal meta‐analysis of pooled data were excluded (e.g., narrative reviews, reviews of qualitative studies).

**Figure 2:**
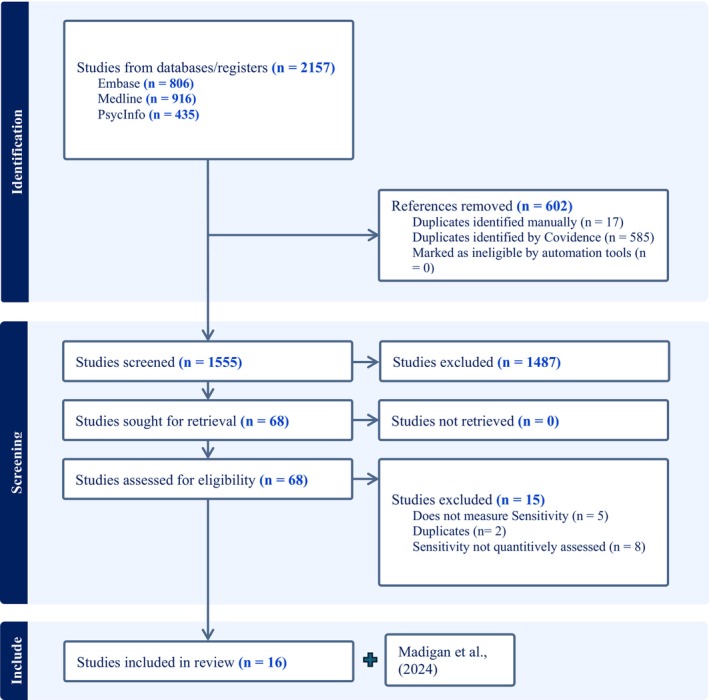
PRISMA flow chart of study selection

Following PRISMA guidelines, all abstracts were uploaded to Covidence and double screened by two independent reviewers (99.5% agreement). Twenty percent of the full text articles were also double coded for the purpose of reliability, with discrepancies resolved via consensus. Agreement across coders was excellent (93% agreement). Sixteen meta‐analyses met inclusion criteria for the present umbrella review (see Figure 2). An additional in‐press meta‐analysis by the senior author was also included in the present review given its relevance resulting in 17 meta‐analyses overall included in this review. This review was registered to Prospero on October 18, 2024 (Prospero ID: CRD42024598684).

### Organizing parenting constructs and child outcomes for analysis

After the review process was complete, we examined the 17 included meta‐analyses and organized them by (1) parenting variable type (i.e., sensitivity or a related construct) and (2) child outcome domain (e.g., attachment, language, cognition, socioemotional, etc.). The purpose of organizing the meta‐analytic literature was to determine whether there was enough consistency in operationalizations of constructs across meta‐analyses, in both the predictor and outcome variables, to conduct quantitative umbrella analyses. The 17 meta‐analyses that met inclusion criteria examined a variety of sensitivity constructs as well as child outcomes. Two meta‐analyses (i.e., McIntosh et al., 2021; Madigan et al., 2019) reported on sensitivity and at least one other construct and were included in both Aim 1 and Aim 2.

#### Sensitivity constructs

Among included meta‐analyses in this umbrella review, 11 explicitly focused on a construct operationalized as sensitivity – though one study did not report primary study data, and the authors were not able to provide it; therefore, it was excluded from analyses. Subsequently, N=10 meta‐analyses were included in the quantitative analyses in Aim 1.

Among the eight meta‐analyses available for Aim 2, the caregiving related constructs included: parental responsivity (N=1 meta‐analysis), parent–child behavioral synchrony (N=1), perceived parental warmth (N=16), observed parental warmth (N=2), caregiving intrusiveness (N=1), parental autonomy granting (N=1), parental behavioral control (N=1), harsh control (N=1), psychological control (N=1), supportive parent–child relationships (N=2), negative parent–child relationships (N=2), cognitive parenting behaviors (e.g., scaffolding; N= 1), negative relationship quality (N=1), and positive relationship quality (N=1). Four meta‐analyses examined multiple constructs of parenting (e.g., Pinquart 2016 examined both autonomy support and warmth). Given the diverse range of constructs among the meta‐analyses included in Aim 2, only narrative results are provided.

#### Child developmental outcomes for Aim 1

Next, we examined the developmental outcomes of the meta‐analyses that were included in Aim 1. The following child outcome constructs were examined: internalizing and externalizing problems (N=2 meta‐analyses), ADHD diagnosis and symptoms (N=1), emotion regulation (N=1), parent–child attachment security (N=6), cognition (N=3), and language skills (N=3).

#### Defining factors

The *metaumbrella* package requires users to format the data very specifically (see Gosling et al., 2023), and because there was a range of developmental outcomes included, we had to organize the outcomes into broad domains (or factors, per *metaumbrella*) for analysis. Two factors were immediately apparent given their consistent definitions across meta‐analyses: parent–child attachment security and language skills. Beyond that, outcomes could be categorized into broadly defined cognitive function and socioemotional problems.

#### Broad cognitive functioning

Consistent with Deneault et al. (2023), we defined cognition as children's cognitive capacities or intelligence. The measures included in this meta‐analysis focused on broad cognitive functioning or intelligence and included direct and parent‐report measures such as the Ages and Stages Questionnaire and standardized tests such as the Stanford‐Binet. Rodrigues et al. (2021) examined cognitive ability, which was defined as the direct testing utilizing standardized assessments of cognitive ability such as the Bayley's Scale of Infant Development. Rodrigues et al. (2021) also examined executive function skills; however, given that this construct very specifically focused on measures of inhibitory control, cognitive flexibility, and working memory, we did not include executive function in the umbrella analysis. Therefore, the cognitive factor only drew upon Deneault et al. (2023) and Rodrigues et al. (2021) studies, including general cognitive skills, ability, and intelligence.

#### Socioemotional problems

The remaining meta‐analyses examined internalizing (i.e., depression, anxiety) and externalizing (i.e., aggression) problems, ADHD diagnoses and symptoms, and emotion regulation. We decided to include these outcome variables into one socioemotional factor given the bidirectional relationship between emotion regulation and symptoms of psychopathology (Dawel et al., 2021), though sensitivity analyses were conducted examining these constructs separately to ensure that this grouping did not affect the overall results.

In sum, for Aim 1, we examined the association between parental sensitivity and four broad factors of development: parent–child attachment quality, child language skills, child broad cognitive functioning, and child socioemotional problems.

### Data extraction and harmonization

#### Data extraction – meta‐analytic level

For Aims 1 and 2, data were extracted based on the characteristics of each meta‐analysis. Data were extracted with the use of a structured protocol. The following variables were extracted: type of sensitivity measure, name of outcome variable (e.g., language, internalizing symptoms), type of outcome variable (e.g., parent, child, or dyadic variable), subtype of outcome variable, total number of studies included (*k*), total *N*, overall effect sizes reported, moderators examined, and results of moderator analyses. Approximately 20% of studies were extracted for reliability – agreement was good (84% agreement).

If meta‐analyses did not report the effect size as a correlation, the effect sizes were converted to bivariate correlations for ease of interpretation using the *esc* package (Lüdecke et al., 2019) in *R*. All meta‐analyses included in Aim 1 reported the meta‐analytic effect size as bivariate correlations, so no conversions were necessary. One meta‐analysis in Aim 2 reported the meta‐analytic effect size as an unstandardized beta – these effect sizes were then converted to bivariate correlations for consistency across studies.

#### Data extraction – primary study level

To conduct a quantitative umbrella analysis, individual effect sizes from each primary study reported within a meta‐analysis must be extracted. Therefore, we extracted all primary study data reported with each of the 10 meta‐analyses included in Aim 1. We also extracted data for moderation analyses, including child age in months, child sex (% male), parent gender (% female), family SES (i.e., low, mid‐high, mixed), or study design (i.e., cross‐sectional vs. longitudinal).

After all primary study effect sizes were extracted, we used the “check data” function in the *metaumbrella* package to identify any duplicate studies across all meta‐analyses. Several meta‐analyses had overlapping effect sizes, and to avoid redundancy, we systematically removed duplicates so a primary study would not be counted multiple times in the umbrella analysis. For example, both Madigan et al. (2024) and Zeegers et al. (2017) included the effect size between sensitivity and attachment reported in Bernier, McMahon, and Perrier (2017). Previous evidence (i.e., Mathes, Klaßen, & Pieper, 2017) has demonstrated that there is a high rate of inconsistency in effect size reporting across meta‐analyses; therefore, when encountering duplicate studies, we strived to include the most accurately reported effect size. If there was a discrepancy in the effect sizes reported across meta‐analyses, effect sizes were cross‐referenced with the original source papers, and the most accurate effect size was included. If there were no discrepancies in the reported effect sizes, the effect size from the most recent meta‐analysis was included.

Additionally, when meta‐analyses reported effect sizes derived from composite measurements (e.g., average of multiple time points), those effect sizes were excluded if non‐composite effect sizes were also available for the same study. However, if only composite effect sizes were reported, these were included in the umbrella analysis. For example, in a recent meta‐analysis (Deneault et al., 2023), three effect sizes were extracted from Lemche, Klann‐Delius, Koch, and Joraschky (2004): caregiver sensitivity to cognition measured at 12 months, at 24 months, and to a composite measure of the 12‐ and 24‐month effect sizes. In this case, we included the two effect sizes that examined sensitivity at 12 and 24 months and excluded the remaining composite effect size.

We did not extract primary study data for the studies in Aim 2, as statistical analyses could not be conducted given the variability in constructs.

#### Study quality

Quality of the included meta‐analyses was evaluated using the National Institutes of Health's Quality Assessment of Systematic Reviews and Meta‐Analyses tool (National Institute of Health, 2021). The assessment tool evaluates whether eight indices of quality are reported in the meta‐analysis, including items such as whether authors provide explicit inclusion and exclusion criteria, whether authors rated primary studies for quality, and whether all included studies were cited. A second independent coder rated 50% of included studies for study quality. Percent agreement between the two coders was excellent (94% proportionate agreement).

### Data analysis

#### Data synthesis

For Aim 1, a random effects meta‐analysis was conducted using the *metaumbrella* (Gosling et al., 2023) package for *R*. The *metaumbrella* package was developed in order to streamline and standardize the umbrella review process. It allows for multiple effect sizes from the same study, so long as authors designate whether the effect sizes are either from independent subgroups (e.g., clinical vs. control) or from different outcome variables measured in the same participants (e.g., attachment measured multiple times). In the case of multiple outcomes, the within‐study correlation between outcomes was set at .50 (the default setting of *metaumbrella*, consistent with Borenstein, Hedges, Higgins, & Rothstein, 2021), though we conducted sensitivity analyses in increments of .10 for the within‐study correlation from .30 to .80. Narrative summary results are presented for Aim 2.

Effect sizes were evaluated based on Funder and Ozer's (2019) criteria for effect sizes in psychological science (very small: *r* = .05–.09, small: *r* = .10–.19, medium/moderate: *r* = .20–.29, large: *r* = .30–.39, very large: *r* = .40 or greater) as well as the attachment field‐specific effect sizes proposed by Schuengel, Verhage, and Duschinsky (2021): small effect size (*r* = .10), medium/moderate effect size (*r* = .20), and large effect size (*r* = .30). The *R* package *ggplot* (Wickham, 2006) was used to visually represent the results of Aim 1.

#### Moderation analyses

Moderators cannot be examined using the *metaumbrella* package. Therefore, all moderation analyses were conducted using *metafor*. Moderators were chosen based on the availability and consistency of coding of variables across meta‐analyses, which ultimately included child age and biological sex, parent gender, family SES, and study design.

#### Sensitivity analyses

To assess the efficacy of the *metaumbrella* package, all analyses were re‐run in *metafor* (Viechtbauer, 2010) in *R*.

## Results

### Study characteristics

The characteristics of meta‐analyses included in Aim 1, including study quality, are outlined in Table 1. Overall, 10 meta‐analyses consisting of 24 pooled effect sizes examined the association between caregiver sensitivity to child development. Six of the 10 meta‐analyses received a perfect score on the quality ratings, 3 out of 10 scored 7/8, and one scored 6/8 (see Table 1). Given the lack of variability in the quality findings, study quality was not examined as a moderator.

Meta‐analyses were then grouped by domain for analyses using the *metaumbrella* package, resulting in four overarching domains: attachment quality, socioemotional problems, cognition, and language skills. Table 2 outlines which meta‐analyses were included in each domain.

**Table 1:** Characteristics of meta‐analyses included in Aim 1 (sensitivity only)

Study	Sensitivity	Outcome variable	Observed sensitivity specified in inclusion criteria?	*k*	*n*	*r*	95% CI	Study quality
Brumariu et al. (2013)	Paternal sensitive caregiving including sensitivity, acceptance, or involvement	Attachment security	No	6	1,674	.45	0.10 to 0.44	8/8
Brumariu et al. (2013)	Maternal sensitive caregiving including sensitivity, acceptance, or involvement	Attachment security	No	7	2041	.28	0.30 to 0.59	8/8
Claussen et al. (2022)	Sensitivity/warmth	Overall ADHD	No	10	NR	−.16	−0.20 to 0.12	6/8
Claussen et al. (2022)	Sensitivity/warmth	Inattention	No	5	NR	−.17	−0.24 to 0.10	6/8
Claussen et al. (2022)	Sensitivity/warmth	Hyperactivity	No	5	NR	−.14	−0.21 to 0.07	6/8
Cooke et al. (2022)	Observed parental sensitivity	Internalizing problems	Yes	69	14,729	−.08	−0.12 to 0.05	7/8
Cooke et al. (2022)	Observed parental sensitivity	Externalizing problems	Yes	94	25,418	−.14	−0.17 to −0.11	7/8
Cossette‐Cote et al. (2022)	Maternal sensitivity	Attachment security	No	7	NR	.47	0.32 to 0.60	8/8
Deneault et al. (2023)	Sensitivity	Attachment security	Yes	24	NR	.24	0.16 to 0.32	8/8
Deneault et al. (2023)	Sensitivity	Cognition	Yes	18	NR	.21	0.13 to 0.29	8/8
Deneault et al. (2023)	Sensitivity	Language outcomes	Yes	12	NR	.13	0.09 to 0.17	8/8
Lucassen et al. (2011)	Paternal sensitivity	Infant‐father attachment	Yes	16	1,355	.12	0.05 to 0.20	7/8
Madigan et al. (2019)	Sensitive responsiveness	Language skills	Yes	36	7,315	.27	0.21 to 0.33	8/8
Madigan et al. (2024)	Parental Sensitivity	Attachment security	Yes	174	22,914	.25	0.22 to 0.28	7/8
Rodrigues et al. (2021)	Paternal sensitivity	Language skills	Yes	9	552	.21	0.13 to 0.29	8/8
Rodrigues et al. (2021)	Paternal sensitivity	Cognitive ability	Yes	9	1,180	.18	0.10 to 0.25	8/8
Rodrigues et al. (2021)	Paternal sensitivity	Cognition (Executive functioning)	Yes	8	1,761	.19	0.09 to 0.28	8/8
Rodrigues et al. (2021)	Paternal sensitivity	Internalizing problems	Yes	8	487	−.02	−0.13 to 0.09	8/8
Rodrigues et al. (2021)	Paternal sensitivity	Emotion regulation	Yes	7	2,634	.22	0.07 to 0.36	8/8
Rodrigues et al. (2021)	Paternal sensitivity	Externalizing problems	Yes	19	1,057	−.08	−0.14 to −0.03	8/8
Zeegers et al. (2017)	Parental sensitivity	Attachment security	Yes	51	6,664	.25	0.20 to 0.31	8/8

*k* = total number of studies, *n* = total number of participants, *r* = correlation coefficient, 95% CI = confidence interval. All effect sizes reported in *r* for ease of comparison – if the source meta‐analysis did not report *r*, effect sizes were converted using the *esc* package. Observed sensitivity = whether the meta‐analysis specified that only observed sensitivity was included.

**Table 2:** Meta‐analyses included in each factor for *metaumbrella* analyses

Attachment quality	Socioemotional functioning	Cognition	Language skills
Brumariu et al. (2013) Cossette‐Côté et al. (2022) Deneault et al. (2023) Lucassen et al. (2011) Madigan et al. (2024) Zeegers et al. (2017)	Claussen et al. (2022) Cooke et al. (2022) Rodrigues et al. (2021)	Deneault et al. (2023) Rodrigues et al. (2021)	Deneault et al. (2023) Madigan et al. (2019) Rodrigues et al. (2021)

Table 2 outlines each meta‐analysis included in the umbrella factors.

### Aim 1: To what extent is caregiver sensitivity meta‐analytically predictive of developmental outcomes?

#### Pooled effect size estimates

**Figure 3:**
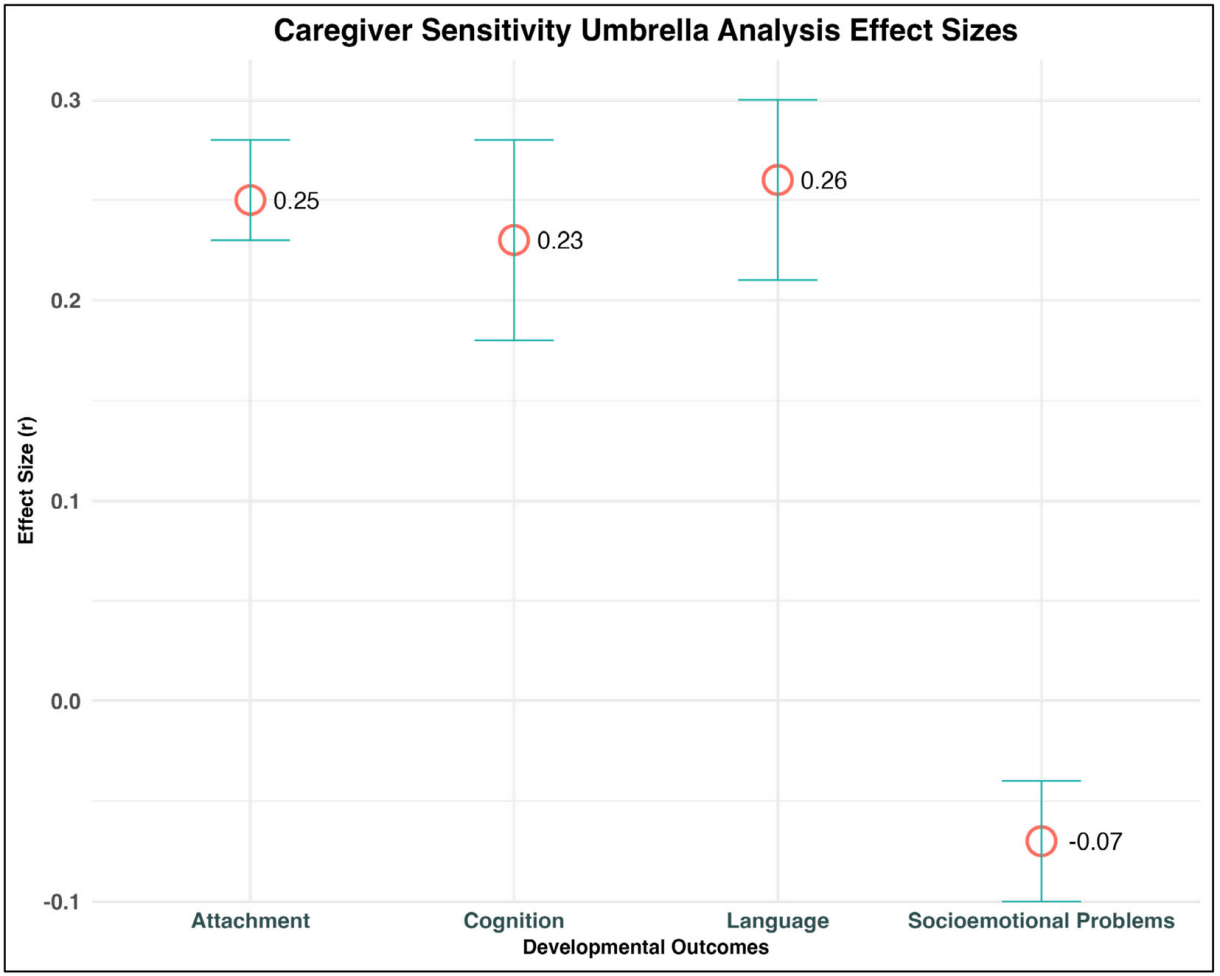
This plot illustrates the umbrella effect sizes between caregiver sensitivity, attachment, cognition, language, and socioemotional outcomes, represented as point estimates with error bars indicating the confidence intervals. The *y*‐axis denotes pooled effect sizes (correlation coefficient)

Results are outlined in Table 3 and depicted in Figure 3.

**Table 3:** Meta‐umbrella results via *metaumbrella*

Factor	*k*	Participant n	Pooled Effect size (*r*)	95% CI	*p* value	*I* ^2^	Egger *p* value
Attachment	253	37,444	.25	0.23 to 0.28	<.001	79.38%	.06
Socioemotional problems	135	33,305	−.07	−0.10 to −.04	<.001	82.17%	.25
Cognition	31	4,740	.23	0.17 to 0.29	<.001	79.34%	<.05
Language	54	11,136	.26	0.21 to 0.30	<.001	80.29%	.15

Factor (specific aspect analyzed), *k*  (number of studies), Participant *n* (total sample size), Pooled effect size (correlation coefficient), 95% CI (confidence interval), *p* value (statistical significance), *I* ^2^ (percentage of variability due to heterogeneity), and Egger's Test *p* value (assessing publication bias).

##### Attachment quality

253 primary studies consisting of 37,444 participants were included in the umbrella analysis. Overall, caregiver sensitivity was moderately associated with attachment quality (*r* = .25, 95% CI [0.23, 0.28]). There was evidence of heterogeneity (*I* ^*2*^ = 79.38%) and only very weak evidence of publication bias (Egger's test *p* value = .06).

##### Socioemotional problems

135 individual studies consisting of 33,305 participants were included in the umbrella analysis. Overall, the magnitude of the association between caregiver sensitivity and socioemotional problems was very small (*r* = −.07, 95% CI [−0.10, −0.04]). There was evidence of heterogeneity (*I* ^2^ = 82.17%) and no evidence of publication bias (Egger's test *p* value = .25). We also examined the effect sizes between caregiver sensitivity and the subdomains of socioemotional problems (see Table S1). Caregiver sensitivity continued to be weakly associated with internalizing symptoms (*r* = −.07, 95% CI [−0.10, −0.03]) and externalizing behaviors (*r* = −.07, 95% CI [−0.10, −0.03]). The association between sensitivity and symptoms of ADHD was higher in magnitude (*r* = −.15, 95% CI [−0.20, −0.15]).

##### Cognition

Thirty‐one individual studies consisting of 4,470 participants were included in the umbrella analysis. Overall, caregiver sensitivity was moderately associated with cognition (*r* = .23, 95% CI [0.17, 0.29]). There was evidence of heterogeneity (*I* ^2^ = 79.34%) and a significant Egger's test did suggest evidence of publication bias (*p* < .05).

##### Language skills

Fifty‐four individual studies consisting of 11,136 participants were included in the umbrella analysis. Overall, caregiver sensitivity was moderately associated with language skills (*r* = .26, 95% CI [0.21, 0.30]). There was evidence of heterogeneity (*I* ^*2*^ = 80.29%) and no evidence of publication bias (Egger's test *p* value = .15).

#### Moderation analyses

An additional advantage of conducting umbrella analyses via *metafor* is the ability to perform moderator analyses, which the current version of the *metaumbrella* is not able to do. We examined whether child age (in months), child sex at birth (% male), parent gender (% female), family SES (i.e., low, mid‐high, mixed), and study design (i.e., cross‐sectional vs. longitudinal) moderated the association between sensitivity and each domain (attachment quality, socioemotional problems, cognition, and language skills). Moderation results are outlined in Tables 4 and 5.

**Table 4:** Continuous moderation results traditional analyses

Factor	*k*	Effect sizes	*r*	95% CI	*t*‐value	*p*
**Attachment**						
Child age	254	331	.00	.00 to .00	1.74	.08
Child sex at birth	193	260	.01	.00 to .01	4.53	<.05
Parent gender	187	245	.00	.00 to .01	0.91	.36
**Socioemotional**						
Child age	134	224	.00	.00 to .00	0.75	.45
Child sex at birth	127	212	.00	.00 to .00	−0.10	.92
Parent gender	106	171	.00	.00 to .00	−1.36	.17
**Cognition**						
Child age	31	36	.00	.01 to .00	−0.43	.67
Child sex at birth	32	36	.00	.01 to .01	0.20	.84
Parent gender	23	27	.13	−.26 to .01	−1.82	.07
**Language**						
Child age	54	66	.00	.00 to .01	0.00	.09
Child sex at birth	54	66	.00	−.01 to .01	0.01	.99

Table 4 presents moderation results for continuous moderators. Factor (specific aspect analyzed), *k* (number of studies), effect sizes, *r* (correlation coefficient), 95% CI (confidence interval range), *t*‐value, and *p* value. The analysis examining parent gender as a moderator of caregiver sensitivity and child language skills was not conducted as all caregivers in the sample were female. Child age in months; Child Sex at Birth = % male children in sample; Parent Gender = % female caregivers in sample.

**Table 5:** Moderation results traditional analyses

	*k*	Effect sizes	*r*	95% CI	*t*‐value	*p*
***Attachment***						
**SES**						.78
Low	32	46	.20	.15 to .26	6.89	
Mid‐high	60	81	.23	.18 to .27	9.68	
Mixed	67	94	.23	.18 to .27	10.54	
**Study design**						.14
Cross‐sectional	117	158	.24	.21 to .27	14.01	
Longitudinal	105	129	.27	.23 to .29	15.16	
***Socioemotional***						
**SES**						.94
Low	22	35	−.05	−.12 to .02	−1.40	
Mid‐high	15	23	−.06	−.15 to .02	−1.43	
Mixed	42	70	−.06	−.11 to −.01	−2.54	
***Cognition***						
**SES**						.41
Low	6	8	.25	.10 to .39	3.33	
Mid‐high	5	5	.15	.00 to .41	1.92	
Mixed	6	6	.29	.16 to .41	4.25	
**Study design**						.95
Cross‐sectional	18	21	.25	.17 to .33	6.27	
Longitudinal	6	6	.26	.16 to .35	5.18	
***Language***						
**SES**						<.05
Low	8	10	.39	.28 to .48	6.75	
Mid‐high	16	19	.20	.11 to .27	4.67	
Mixed	22	26	.27	.20 to .33	7.58	
**Study design**						.43
Cross‐sectional	14	18	.29	.21 to .37	6.74	
Longitudinal	34	40	.26	.20 to .31	8.59	

Factor (specific aspect analyzed), *k* (number of studies), effect sizes, *r (correlation coefficient)*, 95% CI (confidence interval range), *t*‐value, and *p* value. SES: socioeconomic status.

Results demonstrated that child sex moderated the association between caregiver sensitivity and parent–child attachment security, such that samples with a higher percentage of males had stronger associations between sensitivity and attachment, such that for every 1% increase in the percentage of males per sample, the association between sensitivity and attachment increased by .006 (95% CI [.004–.01]). Additionally, SES moderated the association between caregiver sensitivity and child language skills such that the association was much higher in low SES samples (*r* = .39, 95% CI [.28–.48]) compared to mid‐high SES (*r* = .20, 95% CI [.12–.28]) and mixed SES samples (*r* = .27, 95% CI [.20–.33]). No other significant moderation was found.

#### Sensitivity analyses

We conducted sensitivity analyses to examine different levels of assumed within‐study correlations ranging in .10 increments from .30 to .80. Study results did not differ when the within‐level correlations fluctuated (see Table S2). Additionally, we confirmed our umbrella analyses results by running a multilevel random effects meta‐analysis using the *metafor* (Viechtbauer, 2010). We conducted four separate meta‐analyses examining the association between sensitivity and (1) attachment security, (2) socioemotional problems, (3) broad cognitive functioning, and (4) language skills. Results were consistent with the results derived from the *metaumbrella* package (see Table S3), with the exception of some indicators of publication bias. The results of the sensitivity analyses found that the sensitivity‐attachment meta‐analysis demonstrated evidence of publication bias. However, the Egger's test performed using the *metaumbrella* package was marginally significant (*p* = .06). Furthermore, the sensitivity analyses did not find any evidence of publication bias for the sensitivity‐cognitive meta‐analysis (*p* = .48), despite the *metaumbrella* package indicating that there was publication bias.

### Aim 2: How are constructs related to sensitivity (e.g., warmth, control) meta‐analytically associated with developmental outcomes?

The characteristics of meta‐analyses included in Aim 2 are outlined in Table S4. For study quality, seven out of eight of the meta‐analyses received a perfect score on the quality ratings – the remaining study received a score of 7/8 (see Table S4). In this section, we narratively review the meta‐analytic evidence for the association between the broader array of caregiving constructs linked to sensitivity and child outcomes.

#### Attachment

McIntosh, Schnabel, Youssef, and Olsson (2021) examined the meta‐analytic association between caregiving interference/intrusiveness and attachment disorganization in children aged 1–6 years old – their reported effect was moderate in magnitude (*r* = .31, *k* = 2, *n* = 305).

#### Socioemotional

Davis, Bilms, and Suveg (2017) examined the association between parent–child positive behavioral synchrony (regulated, reciprocal, and harmonious interactions) and child self‐regulation in studies utilizing both cross‐sectional and longitudinal designs – their reported effect was moderate in magnitude (*r* = .32, *k* = 10). Children were on average 31.85 months of age at the synchrony assessment and 49.38 months of age at the self‐regulation assessment.

Khaleque (2013) examined the associations between perceived maternal and paternal warmth and several factors of children's psychological adjustment (see Table S4 for effect sizes per each subscale). Children were on average 12 years of age. Perceived maternal and paternal warmth were associated with overall child psychological adjustment (maternal: *r* = .33, *k* = 33, *n* = 7,596; paternal: *r* = .34, *k* = 10, *n* = 2,343). These associations were similar to or larger in size than those previously reviewed for sensitivity, although the reliance on the same informant for measurements of antecedent and outcome means these associations are likely to be upwardly biased.

#### Relational

Schulz et al. (2023) examined overall positive and negative parent–adolescent relationship quality and its association with later peer and romantic relationship quality. Parent–adolescent quality included warmth, responsiveness, and nurturance, as well as measures of conflict and hostility. Overall, supportive parent–adolescent relationship quality was associated with peer (*r* = .18, *k* = 54, *n* = 51,819) and romantic outcomes (*r* = .11, *k* = 38, *n* = 18,763). Negative parent–adolescent relationship quality was also associated with peer (*r* = −.12, *k* = 54, *n* = 51,819) and romantic outcomes (*r* = −.09, *k* = 38, *n* = 18,763). It is notable that the size of these associations are consistently smaller than most of those we have reviewed up to this point (with the exception of sensitivity and socioemotional outcomes). It is unclear whether this reflects the nature of the outcome domains examined or the domain of caregiving.

#### Language

Madigan et al. (2019) examined the meta‐analytic association between parental warmth and child language (average 33.5 months of age). Parental warmth was weakly to moderately associated with child language (*r* = .16, *k* = 13, *n* = 1,961). The association between sensitive responsiveness and language was statistically larger than the association between warmth and language. Additionally, Borairi, Fearon, Madigan, Plamondon, and Jenkins (2021) found that maternal responsivity was moderately associated with child language skills (*r* = .25, *k* = 17, *n* = 6,433) – children were on average 36 months of age.

#### Cognition

Pinquart (2016) examined the meta‐analytic association between several facets of parenting (parental warmth, parental psychological control, parental behavioral control, parental autonomy granting, and parental harsh control) and academic achievement. Children were on average 13.19 years old. Academic achievement was weakly to moderately meta‐analytically associated with parental warmth (*r* = .14, *k* = 308), parental psychological control (*r* = −.11, *k* = 308), parental autonomy granting (*r* = .11, *k* = 308), and parental harsh control (*r* = −.16, *k* = 308). Valcan, Davis, and Pino‐Pasternak (2018) examined the meta‐analytic associations between three aspects of parenting behaviors: positive parenting (e.g., warmth, responsiveness, sensitivity), negative parenting (e.g., control, intrusiveness, detachment), cognitive parenting behaviors (e.g., scaffolding, autonomy support, cognitive stimulation), and executive function up to 8 years of age. All three parenting domains were moderately associated with executive function – specifically positive parenting (*r* = .25, *k* = 41), negative parenting (*r* = −.22, *k* = 41), and cognitive parenting behaviors (*r* = .20, *k* = 41).

## Discussion

The present umbrella review describes and synthesizes the meta‐analytic literature on caregiver sensitivity using an innovative umbrella review methodology. Our results highlight three striking findings. First, based on 473 primary studies (with 86,625 participants) across 35 different countries, there is consistent evidence that caregiver sensitivity is significantly associated with multiple domains of child development. Second, we observed relatively little variation in the strength of the effect sizes between caregiver sensitivity and developmental domains such as attachment, cognition, and language, each of which showed stronger associations compared to socioemotional problems. Third, most of the existing meta‐analytic studies have concentrated on the construct of sensitivity itself, while reviews related to other related constructs, such as warmth, responsivity, and negative parenting, are more disparate and inconsistent. Each of these findings will be discussed in turn, followed by a consideration of evidence gaps and clinical implications.

### The broad developmental reach of caregiver sensitivity

Although caregiver sensitivity originated within the field of attachment, findings from this review indicate that attachment is far from its only developmental correlate. Specifically, the effect sizes for caregiver sensitivity and child language (*r* = .26) and cognition (*r* = .23) were comparable to that for attachment security (*r* = .25). This finding underscores the concept of multifinality – the idea that a single starting point, such as receiving consistently high “doses” of attuned and responsive caregiving, can lead to multiple developmental advantages for children.

If we momentarily assume that these associations are causal, these data suggest that sensitivity acts as a kind of elixir that facilitates child development on multiple fronts. This may be because sensitive responses – characterized by responsivity and context‐appropriateness– provide valuable signals that guide learning or – even more broadly construed – optimize development by providing scaffolding and support and building confidence and skills across multiple domains.

Our findings highlight the possibility that the positive impacts of sensitivity‐focused intervention could extend to a broad range of child outcomes, beyond what might have been the original intended target. Indeed, support for that possibility comes from a review by Jeong, Franchett, Ramos de Oliveira, Rehmani, and Yousafzai (2021). This large meta‐analytic review included trials from a wide range of high‐ and low‐income countries and they found that interventions that focused on promoting responsive caregiving produced reliable and moderate‐to‐large improvements in language (standardized mean difference .31, *k* = 29) and cognitive outcomes (standardized mean difference .38, *k* = 36). Although it is not possible to determine precisely how closely these responsive caregiving interventions targeted sensitivity per se, the similarity between the two constructs makes that more likely than not. Further research to investigate the overlap in intervention elements between attachment‐ and sensitivity‐focused interventions and the broader set of responsive caregiving interventions reviewed by Jeong and colleagues would be invaluable, as would work investigating the role of sensitivity as a mediating mechanism in early child development interventions.

### Differential predictive strength: Cognitive versus socioemotional outcomes

One of the major advantages of an umbrella analysis is that it allows for the examination of multiple outcomes simultaneously – and in the present review, an interesting pattern was revealed. Specifically, the association between sensitivity and socioemotional problems was smaller in magnitude (*r* = −.07) compared to cognitive (*r* = .23), language (*r* = .26), and attachment (*r* = .25) outcomes. This pattern held when we conducted sensitivity analyses to examine individual components of socioemotional problems as well (i.e., ADHD symptoms, internalizing, and externalizing problems).

Given its origin in the field of attachment (generally thought of as in the socioemotional sphere), these findings are intriguing; however, they do align with research from large longitudinal samples examining these constructs simultaneously (Fraley, Roisman, & Haltigan, 2013; Raby, Roisman, & Booth‐LaForce, 2015; Roisman & Fraley, 2012). These studies drew upon three different longitudinal samples and found that caregiver sensitivity was moderate to strongly associated with child academic skills (~*r* = .32–.43) and social competence (~*r* = .14–.27), and weakly with externalizing behavior (*r* = −.11; Roisman & Fraley, 2013; Raby, Roisman, & Booth‐LaForce, 2015). Additionally, a longitudinal intervention study by Stams, Juffer, and Van IJzendoorn (2002) reported that the association between maternal sensitivity and cognitive development (*r* = .33) was larger in magnitude than maternal sensitivity and social development (*r* = .22). Interestingly, this pattern of findings from observational studies is also partially mirrored by the results of intervention trials targeting responsive caregiving: in the review by Jeong and colleagues mentioned above, the average standardized effect size for socioemotional outcomes was .19, compared to .38 for cognitive development and .31 for language.

There are several possible explanations for why caregiver sensitivity might be more strongly associated with cognitive than socioemotional outcomes, in observational and intervention studies alike.

#### Practical and observable factors

It is plausible that cognitive skills may be more inherently obvious and tangible to parents. For example, it may be easier for a parent to help with language learning than to actively promote social competence and coping behaviors, such as empathy, cooperation, or problem‐solving. This may be especially relevant when children enter school and do homework, with which parents can assist. However, even in young children, it is often easier for parents to know how to respond supportively when a child is learning a word or working on a puzzle than when a child is aggressive, anxious, or disobedient. In the former situation, parental and child goals are more likely to align, whereas in the latter, they may diverge. As a result, optimal parental responses in these more challenging contexts are inherently more conditional and uncertain; for example, deciding when to provide support, when to set boundaries, when to intervene, or when to step back.

#### Methodological considerations

Caregiver sensitivity is often assessed in a context where there is only a single, or narrowly constrained, set of circumstances, which has been designed to elicit certain kinds of behaviors and interactions. For example, an interaction may be observed while a child and parent share a picture book together. In such settings, sensitivity often manifests in verbal exchange such as labeling objects or expanding on a child's vocalization and supporting language learning through “serve and return” interactions (Center on the Developing Child at Harvard University, 2025). Similarly, many observations of sensitivity take place in contexts of free play, or toy‐related play, which tend to elicit interactions that center on supporting the child's exploration and learning. Less commonly, sensitivity may be observed in a context where distress is more likely to occur, and indeed, there is evidence that observational contexts like this produce stronger associations with attachment security compared to low‐stress conditions (Leerkes, Weaver, & O'Brien, 2012).

There is thus reason to believe, as well as some evidence, that sensitivity may have domain‐specific effects, but these may be observed not so much in the different character of the behavior, but rather in the observational contexts and developmental tasks to which they are directed. The weaker association between sensitivity and emotional–behavioral problems, relative to language and cognitive outcomes, could be interpreted in this way, in that the context of observation used in research is typically low stress and focused on toy or free play, and may be biased toward parent–child interactions more directly involved in cognitive and language outcomes than socioemotional ones.

Interestingly, this pattern is not mirrored in meta‐analyses on attachment security and child outcomes. There, similar‐sized associations are found with both attachment security and children's social–emotional (externalizing problems: *r* = .15; Fearon, Bakermans‐Kranenburg, van IJzendoorn, Lapsley, & Roisman, 2010, internalizing problems: *r* = .08; Groh, Roisman, van IJzendoorn, Bakermans‐Kranenburg, & Fearon, 2012) and cognitive (*r* = .17; Deneault et al., 2023) outcomes. Roisman and Fraley (2012) have argued that attachment‐related measures are inherently more emotional than sensitivity observations. For example, attachment is often measured through a paradigm that involves separations and reunions, which are designed to elicit distress and thereby tap into the relational dynamics that underlie children's emotional security (Duschinsky, 2020). In contrast, caregiver sensitivity is typically assessed through structured tasks that focus more on free play, problem‐solving behaviors (e.g., tool task), or feeding interactions. As a result, the most common sensitivity tasks may capture less of the interpersonal dynamics that may be relevant for children's emotion regulation, co‐regulation, and social competence. This distinction in measurement may help explain why caregiver sensitivity (but not attachment security) shows relatively weaker associations with children's socioemotional problems compared to cognitive and language outcomes.

#### Theoretical and evolutionary perspectives

Several researchers (Madigan et al., 2019; Roisman & Fraley, 2012; Romeo et al., 2018) have argued that measures of caregiver sensitivity often reflect a parent's ability to foster intellectual engagement within the child's zone of proximal development. Responding to cues may be particularly crucial for guiding learning in this zone of proximal development, whereas other mechanisms (such as modeling or reinforcing negative behavior or overcontrol) may play a larger role in socioemotional outcomes. Several evolutionary theorists (e.g., Tomasello, Carpenter, Call, Behne, & Moll, 2005) provide further support for this view. They have argued that human cognition evolved to prioritize understanding of the intentions and goals of others.

In the context of a parent–child relationship, parents are typically invested in building their child's skills, knowledge, and competencies, which likely requires the parent to understand the child's cognitive state, goals (i.e., what the child is trying to do or say), and abilities, and for the child and parent to dynamically cooperate to help the child learn. Tomasello et al. (2005) argue that this kind of cooperative communication lays the groundwork for the development of social and cognitive skills. Relatedly, Csibra and Gergely (2009) argue that natural selection gave rise to the evolution of a suite of communicative mechanisms that support the rapid and efficient transmission of knowledge (“natural pedagogy” in their terminology) between learners and “teachers.” Central to these mechanisms are what Csibra and Gergely refer to as ostensive cues, which signal and facilitate the learner to fast‐map communicated information from the “teacher.” Sensitive interactions are rich in these kinds of ostensive cues, particularly eye contact, joint attention, and contingency, which might explain their particular role in supporting cognitive and language outcomes.

Others have suggested that caregiver sensitivity reflects the parent's ability to read the child's mental states, which in turn may more directly nurture language and cognitive development (van IJzendoorn et al., 1995). Parental mind‐mindedness, for example, has been linked to children's early language skills and effortful control, which in turn predict later school readiness (Bernier et al., 2017). Prime et al. (2015) introduced the concept of cognitive sensitivity to capture parental sensitivity in learning contexts. Cognitive sensitivity focuses specifically on interactions that have high communicative clarity (e.g., giving clear directions, following rules), mutuality building (e.g., positive feedback, turn‐taking), and mind‐reading (e.g., sensitive to things the other does not understand, responds to nonverbal requests for help). Cognitive sensitivity has been associated with later child cognitive skills (i.e., receptive vocabulary, executive function, theory of mind, and achievement) but has yet to be investigated in relation to behavioral or social outcomes.

#### Direct and indirect pathways

Building off the notion that sensitivity may directly foster language development through responding to cues, cooperative communication, and joint attention, it is possible that the nature of the association between parental sensitivity and language, cognition, and attachment is an indirect association. That is, sensitivity may have cascading effects on behavior problems through a number of intermediate mechanisms (e.g., see Deneault et al., 2023).

There are a number of reasons why it might be appropriate to think of sensitivity as playing an indirect, mediated role in children's emotional and behavioral problems. One arises out of the evidence that child language is an important predictor of behavior problems (Hentges, Devereux, Graham, & Madigan, 2021). Language also appears to act as an important foundation for several other developmental competencies, including emotion recognition, emotion regulation, and the ability to seek social support, all of which may in turn reduce the risk of child behavior problems (Petersen et al., 2013). Similarly, it may be that sensitivity is associated with behavioral problems via its impact on attachment security (Fearon et al., 2010). Thus, the lower effect size for the association between sensitivity and socioemotional outcomes may reflect the fact that its impact is indirect and mediated via child language, cognition, or attachment. This warrants exploration in future research.

#### The role of genetics

Although language and cognitive ability are highly heritable (Plomin et al., 2013; Plomin & Spinath, 2002), Roisman and Fraley (2012) found that in behavior‐genetic models, the phenotypic correlation between sensitivity and academic skills was largely attributable to environmental (shared and nonshared) influences rather than genetic ones. In line with this notion, a sibling comparison study found that the child who received relatively greater maternal sensitivity, compared to their sibling, demonstrated greater growth in receptive language skills over time (Madigan et al., 2023).

Results of a meta‐analysis of twin samples have also revealed that language and cognitive development in infancy are more strongly influenced by the environment and specifically the shared environment (Austerberry, Mateen, Fearon, & Ronald, 2022). Studies on the development of attachment in infancy and early childhood also tend to suggest strong shared environmental influences (Bokhorst et al., 2003; Roisman & Fraley, 2012). By contrast, Austerberry et al. (2022) found that, even in infancy, emotional and behavioral development tended to show quite marked genetic influence and little shared environment. Thus, although the balance of genetic and environmental influences on language, cognition, and attachment shifts over time (see, e.g., Briley & Tucker‐Drob, 2013; Fearon, Shmueli‐Goetz, Viding, Fonagy, & Plomin, 2014), the environment nevertheless plays an important role, particularly in infancy and early childhood, which may help to explain the stronger associations we observed between parental sensitivity and these outcomes.

### Moderators of the association between caregiver sensitivity and child outcomes

We also examined the extent to which demographic and study characteristics moderated the association between caregiver sensitivity and child outcomes. Results revealed that the association between caregiver sensitivity and parent–child attachment was slightly higher in samples with more male children. However, of the six meta‐analyses in this umbrella review, only three reported data on child sex (Cossette‐Côté et al., 2022; Deneault et al., 2023; Madigan et al., 2024). Thus, our results should be interpreted with caution.

We also found that the association between caregiver sensitivity and child outcomes was strongest in samples from low and diverse SES groups compared to mid‐high SES groups – a finding consistent with Madigan et al.'s (2019) meta‐analysis on caregiver sensitivity and child language. This pattern suggests that sensitive parenting may be particularly important for supporting child language skills in lower resourced contexts. This highlights the value of early interventions that prioritize supporting caregiver sensitivity as a mechanism for reducing socioeconomic disparities in developmental outcomes, in line with Heckman's (2006) equation.

### Expanding beyond sensitivity: The additive value of related constructs

The present review focused predominately on caregiver sensitivity as a key dimension of caregiving quality; however, our narrative review also considered a wider range of related caregiving constructs, including measures of warmth, parental involvement, intrusiveness, overprotection, demandingness, control, positive and negative relationships with parents, and overall parent–child relationship quality. With the caveat that there were significant variations in operational definitions of these constructs across meta‐analyses, results revealed that, overall, parenting quality was moderately associated with a variety of developmental outcomes, including cognitive, attachment, and socioemotional problems.

Consistent with De Wolff and Van Ijzendoorn (1997), our quantitative analyses and narrative review demonstrate the importance of caregiving on multiple aspects of development. The breadth of parenting constructs studied by researchers, combined with the general lack of differentiation between them in their associations with child outcomes, highlights the need to disentangle their common and distinct contributions to child outcomes.

It is possible that other constructs of parenting, such as positive parenting or synchrony, may be picking up on processes that are not captured by sensitivity alone (Madigan, Plamondon, & Jenkins, 2025). Furthermore, observational and questionnaire measures of caregiving are often poorly correlated (e.g., Nivison et al., 2021), suggesting that they may capture distinct dimensions of caregiving rather than being directly overlapping constructs. With the emergence of artificial intelligence and machine/deep learning, it may be possible to automate the coding of parent–child interactions (see Weng et al., 2025), which will lessen coder burden and can increase the number of constructs that can be coded from a single interaction (e.g., measuring responsivity, synchrony, and sensitivity simultaneously). These kinds of technologies may also offer the prospect of making new discoveries about parenting processes that have hitherto alluded researchers.

### Gaps in the sensitivity meta‐analytic literature

We found specific gaps in the literature on sensitivity and cognitive and socioemotional development, which related primarily to measurement and scope. Specifically, cognition was typically assessed through measures of academic achievement and broad cognitive functioning, leaving other important aspects, such as theory of mind, creativity, and problem solving, largely unexplored. The primary constructs within the socioemotional domain that have been examined in the meta‐analytic literature are attachment security, internalizing, and externalizing behaviors. There has been far less focus meta‐analytically on other aspects of socioemotional functioning – such as social competence, emotional expression, and coping. Future meta‐analytic research should expand the scope of inquiry to include these diverse aspects of cognition and socioemotional development so that a more comprehensive understanding of how caregiver sensitivity influences cognitive and socioemotional outcomes can be achieved.

Important methodological questions about sensitivity also remain underexplored. In particular, little is known about how differences in measurement and construct definition may influence the observed strength of these associations. Future meta‐analyses should examine whether the associations between caregiving and developmental outcomes vary (1) depending on the assessment type (i.e., observation vs. questionnaire) and (2) depending on the construct (e.g., sensitivity vs. related constructs). To our knowledge, there has been little to no meta‐analytic work investigating these potential methodological factors, with the exception of the meta‐analysis by Madigan et al. (2019), which found that the effect size between caregiver sensitivity and language skills was statistically higher than that of the effect size between caregiver warmth and language skills. Findings such as these can help to pinpoint targets of intervention that may have the greatest impact on developmental outcomes.

Another critical gap is the examination of the construct of sensitivity across contexts, regions, and cultures, as well as across caregivers. This is especially important to consider given that some have claimed Ainsworth's scale to be ethnocentric given its focus on child autonomy (i.e., that coding is largely focused on the child initiating cues for attention; Duschinsky, 2020), as well as limited to primary caregivers as a source of sensitive caregiving. Although there was some variation in the primary studies included in the meta‐analyses, they  overwhelmingly were conducted in North America (see Table S5).

The universal applicability of caregiver sensitivity has long been debated in the field. Most who support the universality of sensitivity are focused on the notion that caregivers who are responsive to their child's needs will foster more general wellbeing in their infant, whereas those who critique the universality of sensitivity are largely focused on the measurement of specific sensitive behaviors (see Mesman et al., 2018). For example, in some communities in Kenya, parents rarely respond to nondistress vocalizations, which may be seen as insensitive care in Western societies (see Mesman et al., 2018). Furthermore, it is often the case that the responsibility of caregiving is shared across several individuals, including grandparents, siblings, and more (Mesman et al., 2018) – therefore, it is important to understand how sensitive caregiving in a cumulative setting may be important to the child's well‐being. For example, Mesman et al. (2018) compared videos of observed sensitivity across the Philippines, Republic of Congo, and Mali and found that Ainsworth's sensitivity scale was actually quite suitable for measuring sensitivity across cultural and familial contexts, as the scale allows for “culture‐specific behavioral manifestations that serve the universal function of making sure that infants receive what they need to survive and become adaptive members of their community” (Mesman et al., 2018, p. 846).

Although there have been quite a few preliminary studies that have focused on the measurement of sensitivity across cultures and contexts, this is rarely a focus at the meta‐analytic level. Most meta‐analyses have examined the region where preliminary studies have been conducted as a moderator, but this is often the only aspect of context that is assessed (Madigan et al., 2023). Therefore, it is important that the cultural applicability, beyond only the region in which data were collected, be considered when examining caregiver sensitivity in a meta‐analytic context.

Last, in order to promote caregiver sensitivity, it is important to understand under which conditions parents are sensitive or insensitive (i.e., identifying the antecedents of caregiver sensitivity). Several meta‐analyses have been conducted examining factors that may predict caregiver sensitivity, including sociodemographic risk, parental substance abuse, and parental symptoms of psychopathology, but an umbrella review of the antecedents of sensitivity is still needed to integrate these individual meta‐analyses.

### Practical implications

Although sensitivity is considered the gold standard measure of observed parenting, at least in early development, in practical terms the application of sensitivity has faced challenges in terms of scalability and affordability, particularly in low‐resourced settings. Measuring sensitivity often necessitates controlled, videotaped interactions in research (or home) contexts, which are later assessed by trained coders who extensively review the observation. This approach requires substantial resources, which are often not feasible in large‐scale longitudinal studies or public health interventions, community‐based settings, and in many rural or global contexts (e.g., low‐middle income countries [LMIC]). Therefore, it is critical to adapt measures of sensitivity to be easier and more efficient to administer, while still maintaining their validity (Forrer, Oosterman, Tharner, & Schuengel, 2024).

Attachment‐based interventions were designed to improve caregiver sensitivity with the goal of fostering secure parent–child attachment relationships (e.g., Attachment Biobehavioral Catch up Intervention; Dozier, Bernard, Roben, Steele, & Steele, 2017; VIPP‐SD; O'Hara et al., 2019). These interventions have been studied extensively across diverse populations, and many of the effective programs share core strategies such as psychoeducation about sensitive interactions, video feedback, and commenting on parents' sensitive behaviors through video or “in the moment” feedback.

Bakermans‐Kranenburg et al. (2003) conducted a meta‐analysis on the efficacy of attachment‐based interventions and found that randomized interventions were effective at improving sensitive parenting (*r* = .16) and infant attachment security (*r* = .10). Beyond attachment security, the Attachment Biobehavioral Catch‐up Intervention has yielded broader benefits beyond attachment, including reduced cortisol levels, improved child language, self‐regulation, executive functioning, and peer relations, as well as more optimal brain and neural development. Improvements in some of these domains have been sustained 3–8 years after the intervention (Dozier & Bernard, 2023). The data from sensitivity‐focused interventions suggest that benefits from behavioral problems are likely weaker (Ramchandani et al., 2017), but this would also seem consistent with the findings in this review, in that effect sizes for the association between sensitivity and emotional and behavioral problems were considerably smaller than for attachment, cognitive, or language outcomes.

Although the present review did not examine intervention data, the results of this review may be beneficial in guiding the design of intervention evaluations. For example, interventions focused on improving sensitivity should examine cognitive and language outcomes in addition to socioemotional outcomes. An umbrella review examining interventions focused on improving caregiver sensitivity would be an important and timely addition to the literature (though see Jeong et al., 2021).

It is critical to understand the mechanism linking sensitivity and behavior problems, especially given that many interventions targeting these constructs have had limited success in meaningfully improving children's socioemotional outcomes (but see van IJzendoorn et al., 2023). For example, a long‐term follow‐up of the Video Feedback Intervention to Promote Positive Parenting and Sensitive Discipline (VIPP‐SD) found no evidence of the intervention on child behavior problems compared with a control or a no intervention group (SMD 0.04, 95% CI −0.33 to 0.42, very low‐certainty evidence; O'Hara et al., 2019). Addressing these gaps will be essential for refining intervention strategies to create more enduring change across various aspects of children's development.

### Limitations

One of the major difficulties of conducting an umbrella review on this important topic is the variability in how authors operationalized caregiving. This inconsistency across meta‐analyses can limit the synthesis of findings. Although we set a consistent operational definition of sensitivity for Aim 1, there may still have been variation in how sensitivity was defined across meta‐analyses, and findings should be interpreted in light of this limitation. For example, some meta‐analyses only examined observed sensitivity (e.g., Cooke et al., 2022; Madigan et al., 2024), whereas other meta‐analyses included a variety of measures assessing sensitivity, whether through observation or questionnaire. The umbrella review method was originally created to integrate the evidence of treatment comparisons for specific medical conditions within the biomedical field (e.g., treatment for smoking cessation; Ioannidis, 2009). Operational definitions of conditions within the medical field may be more homogeneous across studies given the criteria set by the World Health Organization's International Classification of Diseases (Rodríguez‐Marín, 2004). In contrast, developmental science often lacks such standardized operational definitions. Moreover, although we were able to examine some moderators, not all meta‐analyses consistently reported the same variables across meta‐analyses, and these findings should be interpreted in light of these limitations.

Our findings do not allow us to make claims regarding the merits of measuring sensitivity observationally versus other methods (e.g., questionnaire), nor do they allow us to make claims about sensitivity versus related constructs (e.g., warmth, hostility, control). That being said, in developmental science, observed parenting (vs. parent or child report via questionnaires) is regarded as the “gold standard” approach (Booth‐LaForce et al., 2014), in part due to lack of bias, objectivity, and the ability to identify behaviors that parents may not be aware of. However, as children age, their perception of the parent–child relationship may provide insight into the quality of the relationship that cannot be gleaned from observations (Nivison et al., 2021), and parenting questionnaires do provide important additional insights, particularly for low frequency but important behaviors (e.g., harsh discipline).

Additionally, a limitation of the present umbrella review is that the findings are correlational in nature, so no definitive causal claims can be made about the predictive significance of caregiver sensitivity. For more causal conclusions, it is helpful to turn to the experimental literature, which suggests that interventions aimed at enhancing caregiver sensitivity can lead to changes in child outcomes (Bakermans‐Kranenburg et al., 2003; Sokolovic, Rodrigues, Tricco, Dobrina, & Jenkins, 2021).

Furthermore, this review assumes a mechanism of influence from parents to children. It has been shown, however, that infant characteristics shape their caregiver's ability to provide sensitive care (Beebe et al., 2010; Browne et al., 2018). Rarely are designs utilized in the sensitivity area that could reveal the directionality of child and parent effects (e.g., cross‐lagged, panel models; exceptions include Madigan et al., 2023; Zvara, Sheppard, & Cox, 2018) or using genetically sensitive designs. As directions of influence are likely to vary across developmental periods and child outcomes, this is an important area for future investigation.

Also relevant to directions of influence is the role of the family system in individual sensitivity. The Social Relations Model provides a design that can separate the extent to which sensitivity is attributable to the individual, the unique combination of individuals in a dyad (direct experience in a relationship), or exposure to dyads in which the individual is not a member (family effect, Sokolovic et al., 2021). The family effect accounts for 14% of the variance in sensitivity. This family process reflects mechanisms of observational learning (or contagion), but it excludes direct experience in a relationship. With such a large family effect on sensitivity, limiting our focus to the caregiver–child dyad is no longer tenable. This, of course, gets to the core issue that observational studies generally lack the ability to make strong causal inferences. Combinations of methods (instrumental variables/Mendelian randomization, longitudinal approaches, and trials) will be important to triangulate evidence on causation. Nevertheless, the success of intervention programs generally lends support to the causal role of caregiver sensitivity in broad terms.

Lastly, although 35 countries are represented, 60% of studies within the 17 meta‐analyses took place in North America (see Table S5 for a full geographical breakdown). Though interventions promoting sensitive caregiving interactions implemented in global contexts, particularly in LMIC, have demonstrated some efficacy in improving parenting quality and child developmental outcomes (Bakermans‐Kranenburg et al., 2003; Jeong et al., 2021; Landry et al., 2021; Sokolovic et al., 2021).

## Conclusion

The present review highlights the importance of caregiver sensitivity and related constructs of parenting in the context of development. Caregiver sensitivity was consistently and strongly associated with multiple factors of development, including attachment quality, as well as cognition and language skills, and to a lesser extent, socioemotional problems. This umbrella review reinforces the critical role of caregiver sensitivity in public health campaigns, such as those led by UNICEF and the Harvard Center on the Developing Child. Although there are limitations to an umbrella design in developmental science and the present findings should not be taken as gospel, this review has provided support for the legacy of caregiver sensitivity on children's developmental health.

## Trial registration

This review was registered to Prospero on October 18, 2024 (Prospero ID: CRD42024598684; https://www.crd.york.ac.uk/PROSPERO/view/CRD42024598684).

## Ethical considerations

Consent procedure and REB approval were not required for the present study as the study only utilizes archival data.

**Key points**

**What is known?**

- Previous meta‐analyses have shown that caregiver sensitivity is a predictor of various child developmental outcomes, including attachment security, mental health, and skill acquisition. However, these findings have remained fragmented, as each meta‐analysis typically focuses on a single outcome, without integrating results across other domain of child development and functioning.

**What is new?**

- The present study conducted an umbrella review (i.e., systematic review of reviews) of the available meta‐analytic literature on the association between caregiver sensitivity and related constructs (i.e., warmth, responsivity, and negative parenting) and child developmental outcomes.

**What is relevant?**

- Results showed caregiver sensitivity was consistently linked to child attachment security, language skills, and cognition and weakly associated with socioemotional problems.
- This evidence suggests that caregiver sensitivity may be an important focus of prevention and intervention efforts to enhance child development.

## Supporting information


**Appendix S1.** Search strategy.
**Table S1.** Running socioemotional disentangled via meta‐umbrella.
**Table S2.** Meta‐umbrella results via meta‐umbrella at different levels of within‐study correlation between outcomes.
**Table S3.** Traditional analyses via metaphor.
**Table S4.** Characteristics of meta‐analyses included in Aim 2 (other related constructs only).
**Table S5.** Geographical distribution of primary studies included in Aim 1.

## Data Availability

These analyses were preregistered, and the data used in this study are available on OSF: https://osf.io/j9su5/?view_only=2a9b9e9f464a4606ba8ede6dfe02299e.
