# Supplementary material for: Annual Research Review: The role of caregiver sensitivity in children's developmental outcomes – an umbrella review
Source: J Child Psychol Psychiatry. 2026 Jan 7;67(4):486–507. doi: 10.1111/jcpp.70087 (PMC13036395; doi:10.1111/jcpp.70087)
Supplement: Supplementary file 1 — Appendix S1. Search strategy. Table S1. Running socioemotional disentangled via meta‐umbrella. Table S2. Meta‐umbrella results via meta‐umbrella at different levels of within‐study correlation between outcomes. Table S3. Traditional analyses via metaphor. Table S4. Characteristics of meta‐analyses included in Aim 2 (other related constructs only). Table S5. Geographical distribution of primary studies included in Aim 1. [file JCPP-67-486-s001.docx]

**Annual Research Review: The Role of Caregiver Sensitivity in Children’s Developmental Outcomes: An Umbrella Review**

**Supporting Information**

**Appendix S1. Search Strategy**

Search term:

Database: Embase <2010 to 2024 January 12>

Search Strategy:

1 parental behavior/ or maternal behavior/ or paternal behavior/ (28267)

2 child parent relation/ or father child relation/ or mother child relation/ (92697)

3 (maternal* or mother* or parent* or paternal* or father*).mp. (1389825)

4 or/1-3 (1389825)

5 (sensitiv* or responsive* or mutual* or synchron*).mp. (3181667)

6 4 and 5 (102054)

7 limit 6 to (consensus development or meta analysis or "systematic review") (2665)

8 meta analysis/ or network meta-analysis/ (304495)

9 "meta analysis (topic)"/ or "systematic review (topic)"/ (74485)

10 practice guideline/ or consensus development/ (593462)

11 "review"/ or "systematic review"/ (3212690)

12 systematic review*.mp. (572724)

13 knowledge synthes*.mp. (662)

14 scoping review*.mp. (26304)

15 cochrane*.mp. (190936)

16 guideline*.mp. (1021085)

17 meta-analys*.mp. (463834)

18 metaanalys*.mp. (13822)

19 ((umbrella or realist or narrative or qualitative or quantitative) adj1 (review* or synthes*)).mp. (54076)

20 or/8-19 (4288503)

21 6 and 20 (11541)

22 7 or 21 (11541)

| **Table S1.** Running socioemotional disentangled via metaumbrella | | | | | | | |
| --- | --- | --- | --- | --- | --- | --- | --- |
| **Factor** | ***k*** | **Participant *n*** | **Pooled Effect Size (*r*)** | **CI** | **P value** | **I^2^** | **Egger P value** |
| Internalizing | 75 | 15,554 | -.07 | -.10 to -.03 | <.001 | 74.96% | .13 |
| Externalizing | 120 | 29,576 | -.07 | -.10 to -.03 | <.001 | 87.79% | .84 |
| ADHD | 10 | 2,475 | -.15 | -.20 to -.11 | <.001 | 18.87% | .28 |

*Note.* Supplemental Table 1 displays the sensitivity analyses examining the components of socioemotional functioning separately. Effect sizes are reported in the expected directions. Factor (specific aspect analyzed), *k* (number of studies), Participant *n* (total participant sample size), Pooled Effect Size (correlation), CI (confidence interval), P value (statistical significance), I² (percentage of variability due to heterogeneity), and Egger P value (assessing publication bias). Emotion regulation was not analyzed separately as only one meta-analysis examined that association and therefore did not meet criteria to be included in an umbrella analysis as its own factor. Rodrigues et al., 2021 reported that the association between paternal sensitivity and emotion regulation was *r* = .22 [.07 to .36].

| **Table S2.** Meta-Umbrella Results via metaumbrella at different levels of within-study correlation between outcomes | | | |
| --- | --- | --- | --- |
| **Factor** | **Effect Size (*r*)** | **CI** | ***p* value** |
| Within-study correlation: .30 | | | |
| Attachment | .25 | .23 to .28 | <.001 |
| Socioemotional | -.07 | -.10 to -.04 | <.001 |
| Cognition | .23 | .17 to .29 | <.001 |
| Language | .26 | .21 to .30 | <.001 |
| Within-study correlation: .40 | | | |
| Attachment | .25 | .23 to .28 | <.001 |
| Socioemotional | -.07 | -.10 to -.04 | <.001 |
| Cognition | .23 | .17 to .29 | <.001 |
| Language | .26 | .21 to .30 | <.001 |
| Within-study correlation: .60 | | | |
| Attachment | .25 | .23 to .28 | <.001 |
| Socioemotional | -.07 | -.10 to -.04 | -.070 |
| Cognition | .23 | .17 to .29 | <.001 |
| Language | .26 | .21 to .30 | <.001 |
| Within-study correlation: .70 | | | |
| Attachment | .25 | .23 to .28 | <.001 |
| Socioemotional | -.07 | -.10 to -.04 | <.001 |
| Cognition | .23 | .17 to .29 | <.001 |
| Language | .26 | .21 to .30 | <.001 |
| Within-study correlation: .80 | | | |
| Attachment | .25 | .23 to .28 | <.001 |
| Socioemotional | -.07 | -.10 to -.04 | <.001 |
| Cognition | .23 | .17 to .29 | <.001 |
| Language | .26 | .21 to .30 | <.001 |

| **Table S3.** Traditional Analyses via metafor | | | | | | | |
| --- | --- | --- | --- | --- | --- | --- | --- |
| **Factor** | ***k*** | **Participant *n*** | **Pooled Effect Size (*r*)** | **95% CI** | **P value** | **I^2^** | **Egger’s *p* value** |
| Attachment | 253 | 37,444 | .25 | .22 to .28 | <.001 | 76.44% | >.01 |
| Socioemotional | 135 | 33,305 | -.07 | -.10 to -.04 | <.001 | 81.81% | .84 |
| Cognition | 31 | 4,740 | .23 | .17 to .30 | <.001 | 70.66% | .48 |
| Language | 54 | 11,136 | .26 | .21 to .30 | <.001 | 82.70% | .67 |

*Note.* Traditional analyses via metafor for various factors in the meta-analysis, including: Factor (specific aspect), *k* (number of studies), Participant *n* (total participant sample size), Effect Size (*r* - strength and direction of relationship), 95% CI (confidence interval), *p* value (statistical significance), I² (variation due to heterogeneity), and Egger’s *p* value (assessing publication bias)

| **Table S4.** Characteristics of meta-analyses included in Aim 2 (other related constructs only) | | | | | | |  |
| --- | --- | --- | --- | --- | --- | --- | --- |
| **Study** | **Sensitivity** | **Outcome variable** | ***k*** | ***n*** | ***r*** | **95% CI** | **Study Quality** |
| Borairi et al., 2021 | Maternal Responsivity | Language | 17 | 6433 | .25 | .19, .31 | 8/8 |
| Davis, 2017 | Parent–Child Positive Behavioral Synchrony | Self-Regulation | 10 | NR | .32 | .24, .40 | 7/8 |
| Khaleque, 2013 | Perceived Maternal Warmth | Psychological Adjustment | 33 | 7596 | .33 | NR | 7/8 |
| Khaleque, 2013 | Perceived Maternal Warmth | Child Hostility Aggression | 25 | 6716 | -.26 | NR | 7/8 |
| Khaleque, 2013 | Perceived Maternal Warmth | Child Independence | 28 | 7154 | .27 | NR | 7/8 |
| Khaleque, 2013 | Perceived Maternal Warmth | Child Positive Self-Esteem | 33 | 7894 | .32 | NR | 7/8 |
| Khaleque, 2013 | Perceived Maternal Warmth | Child Positive Self-Adequacy | 38 | 6517 | .29 | NR | 7/8 |
| Khaleque, 2013 | Perceived Maternal Warmth | Child Emotional Responsiveness | 38 | 8120 | .37 | NR | 7/8 |
| Khaleque, 2013 | Perceived Maternal Warmth | Child Emotional Stability | 28 | 7215 | .20 | NR | 7/8 |
| Khaleque, 2013 | Perceived Maternal Warmth | Child Positive Worldview | 35 | 6208 | .34 | NR | 7/8 |
| Khaleque, 2013 | Perceived Paternal Warmth | Psychological Adjustment | 10 | 2343 | .34 | NR | 7/8 |
| Khaleque, 2013 | Perceived Paternal Warmth | Child Hostility Aggression | 11 | 4191 | -.28 | NR | 7/8 |
| Khaleque, 2013 | Perceived Paternal Warmth | Child Independence | 8 | 3982 | .27 | NR | 7/8 |
| Khaleque, 2013 | Perceived Paternal Warmth | Child Positive Self-Esteem | 10 | 4156 | .34 | NR | 7/8 |
| Khaleque, 2013 | Perceived Paternal Warmth | Child Positive Self-Adequacy | 9 | 2270 | .32 | NR | 7/8 |
| Khaleque, 2013 | Perceived Paternal Warmth | Child Emotional Responsiveness | 9 | 3967 | .36 | NR | 7/8 |
| Khaleque, 2013 | Perceived Paternal Warmth | Child Emotional Stability | 6 | 2890 | .19 | NR | 7/8 |
| Khaleque, 2013 | Perceived Paternal Warmth | Child Positive Worldview | 9 | 3967 | .35 | NR | 7/8 |
| Madigan et al., 2019 | Warmth | Language | 13 | 1961 | .16 | .09, .21 | 8/8 |
| McIntosh et al., 2021 | Caregiving Intrusiveness | Attachment Disorganization | 2 | 305 | .31 | .19, .42 | 8/8 |
| Pinquart, 2016 | Parental Autonomy Granting | Academic Achievement | 308 | NR | .11 | .08, .14 | 8/8 |
| Pinquart, 2016 | Parental Behavioral Control | Academic Achievement | 308 | NR | .11 | .09, .12 | 8/8 |
| Pinquart, 2016 | Parental Harsh Control | Academic Achievement | 308 | NR | -.16 | -.20, -.12 | 8/8 |
| Pinquart, 2016 | Parental Psychological Control | Academic Achievement | 308 | NR | -.11 | -.13, -.10 | 8/8 |
| Pinquart, 2016 | Parental Warmth | Academic Achievement | 308 | NR | .14 | .12, .15 | 8/8 |
| Schulz et al., 2023 | Supportive Parent–Adolescent Relationships | Peer Outcomes | 54 | 51891 | .18 | NR | 8/8 |
| Schulz et al., 2023 | Negative Parent–Adolescent Relationships | Peer Outcomes | 54 | 51891 | -.12 | NR | 8/8 |
| Schulz et al., 2023 | Supportive Parent–Adolescent Relationships | Romantic Outcomes | 38 | 18763 | .11 | NR | 8/8 |
| Schulz et al., 2023 | Negative Parent–Adolescent Relationships | Romantic Outcomes | 38 | 18763 | -.09 | NR | 8/8 |
| Valcan et al., 2018 | Cognitive Parenting Behaviors (Scaffolding, Autonomy Support, Cognitive Stimulation) | Executive Function | 41 | NR | .20 | .16, .26 | 8/8 |
| Valcan et al., 2018 | Negative (Control, Intrusiveness, Detachment) | Executive Function | 41 | NR | -.22 | -.27, -.17 | 8/8 |
| Valcan et al., 2018 | Positive (Warmth, Responsiveness, Sensitivity) | Executive Function | 41 | NR | .25 | .20, .29 | 8/8 |

*Note. k* = total number of studies, *n* = total number of participants, *r* = correlation coefficient, 95% CI = Confidence Interval, NR = Not reported by authors, CD = not enough information reported to convert effect size. All effect sizes reported in *r* for ease of comparison—if source meta-analysis did not report *r,* effect sizes were converted using the *esc* package

|  | | | |  |  |  |  |  |  |  |
| --- | --- | --- | --- | --- | --- | --- | --- | --- | --- | --- |
| **Table S5.** Geographical Distribution of Primary Studies included in Aim One | | | | | | | | | | |
| **Brumariu 2021** | **Claussen 2022** | **Cooke 2022** | **Cossette-Cote 2012** | | **Deneault 2023** | **Lucassen 2011** | **Madigan 2019** | **Madigan 2024** | **Rodrigues 2021** | **Zeegers 2017** |
| Not Reported for sensitivity to attachment ES | UK: 3  USA: 3  Sweden: 1  New Zealand: 1  Netherlands: 1  Germany: 1 | North America: 80  Europe: 19  South America: 1  Oceania: 3  Asia: 1  Middle East: 2  Multiple: 1 | USA: 3  Canada: 1  Korea: 1  Israel: 1  Netherlands: 1 | | Australia: 1  Belgium: 2  Canada: 18  Chile: 3  Germany: 3  Greece: 1  Israel: 4  Italy: 3  Netherlands: 7  Spain: 2  Switzerland: 1  Uganda: 1  UK: 8  USA: 75 | Not Reported | Not Reported | Australia: 2  Canada: 20  Chile: 2  China: 1  Colombia: 2  Finland: 1  France: 1  Germany: 7  Germany & Russia: 1  India: 1  Indonesia: 1  Israel: 8  Italy: 4  Japan: 7  Lithuania: 1  Mali: 1  Mexico: 2  Netherlands: 24  Norway: 1  Peru: 1  Portugal: 6  Romania: 1  Singapore: 2  South Africa: 3  Spain: 1  Sweden: 1  Switzerland: 1  Thailand: 1  UK: 8  UK & Netherlands: 2  USA: 124 | Australia: 1  Canada: 6  Netherlands: 2  New Zealand: 2  Portugal: 1  UK: 6  USA: 23 | Canada: 7  Chile: 1  China: 1  Germany: 2  Indonesia: 1  Israel: 2  Italy: 3  Japan: 1  Korea: 2  Mali: 1  Norway: 1  Portugal: 2  Puerto Rico/USA: 1  South Africa: 1  Thailand: 1  Netherlands: 1  Netherlands/UK: 1  UK: 1  USA: 19  USA & Colombia: 1 |
| Not Reported | 30% North American | 75% North American | 57% North American | | 72% North American | Not Reported | Not Reported | 61% North American | 71% North American | 54% North American |

**References included in Umbrella Review**

* Study included in Aim 1

† Study included in Aim 2

† Borairi, S., Fearon, P., Madigan, S., Plamondon, A., & Jenkins, J. (2021). A mediation meta‐analysis of the role of maternal responsivity in the association between socioeconomic risk and children’s language. *Child Development*, *92*(6), 2177–2193. https://doi.org/10.1111/cdev.13695

* Brumariu, L. E., Obsuth, I., & Lyons-Ruth, K. (2013). Quality of attachment relationships and peer relationship dysfunction among late adolescents with and without anxiety disorders. *Journal of Anxiety Disorders*, *27*(1), 116–124. https://doi.org/10.1016/j.janxdis.2012.09.002

* Claussen, A., Holbrook, J., Hutchins, H., Robinson, L., Bloomfield, J., Meng, L., Bitsko, R., O’Masta, B., Cerles, A., Maher, B., Rush, M., & Kaminski, J. (2022). All in the Family? A Systematic Review and Meta-analysis of Parenting and Family Environment as Risk Factors for Attention-Deficit/Hyperactivity Disorder (ADHD) in Children. *Prevention Science : The Official Journal of the Society for Prevention Research (No Pagination), 2022 Date of Publication: 19 Apr 2022*, no pagination. https://doi.org/10.1007/s11121-022-01358-4

*Cooke, J. E., Deneault, A., Devereux, C., Eirich, R., Fearon, R. M. P., & Madigan, S. (2022). Parental sensitivity and child behavioral problems: A meta‐analytic review. *Child Development*, *93*(5), 1231–1248. https://doi.org/10.1111/cdev.13764

* Cossette-Côté, F., Bussières, E. L., & Dubois-Comtois, K. (2022). The association between maternal sensitivity/availability and attachment in children with autism Spectrum disorder: A systematic review and Meta-analysis. *Current Psychology*, *41*(11), 8236–8248. https://doi.org/10.1007/s12144-021-02227-z

† Davis, C. A. (2017). Social Media Technologies’ Influence on Adolescent Social/Emotional Development Via Attachment. *ProQuest Dissertations and Theses*. https://ezproxy.lib.ucalgary.ca/login?qurl=https://www.proquest.com/docview/1930669825?accountid=9838&bdid=68912&_bd=Nh0ADc3jCDZAIyl9WL8Ip3m4exo%3D

* Deneault, A.-A., Duschinsky, R., Van IJzendoorn, M. H., Roisman, G. I., Ly, A., Fearon, R. M. P., & Madigan, S. (2023). Does child-mother attachment predict and mediate language and cognitive outcomes? A series of meta-analyses. *Developmental Review*, *70*, 101093. https://doi.org/10.1016/j.dr.2023.101093

† Khaleque, A. (2013). Perceived Parental Warmth, and Children’s Psychological Adjustment, and Personality Dispositions: A Meta-analysis. *Journal of Child and Family Studies*, *22*(2), 297–306. https://doi.org/10.1007/s10826-012-9579-z

* Lucassen, N., Tharner, A., Van IJzendoorn, M. H., Bakermans-Kranenburg, M. J., Volling, B. L., Verhulst, F. C., Lambregtse-Van Den Berg, M. P., & Tiemeier, H. (2011). The association between paternal sensitivity and infant–father attachment security: A meta-analysis of three decades of research. *Journal of Family Psychology*, *25*(6), 986–992. https://doi.org/10.1037/a0025855

* Madigan, S., Deneault, A.-A., Duschinsky, R., Bakermans-Kranenburg, M. J., Schuengel, C., Van IJzendoorn, M. H., Ly, A., Fearon, R. M. P., Eirich, R., & Verhage, M. L. (2024). Maternal and paternal sensitivity: Key determinants of child attachment security examined through meta-analysis. *Psychological Bulletin*, *150*(7), 839–872. https://doi.org/10.1037/bul0000433

*† Madigan, S., Prime, H., Graham, S. A., Rodrigues, M., Anderson, N., Khoury, J., & Jenkins, J. M. (2019). Parenting Behavior and Child Language: A Meta-analysis. *Pediatrics*, *144*(4), e20183556. https://doi.org/10.1542/peds.2018-3556

† McIntosh, J. E., Schnabel, A., Youssef, G. J., & Olsson, C. A. (2021). Preconception and perinatal predictors of offspring attachment disorganization: Advancing the replicated evidence. *Development and Psychopathology*, *33*(1), 240–251. https://doi.org/10.1017/S095457941900172X

† Pinquart, M. (2013). Do the Parent–Child Relationship and Parenting Behaviors Differ Between Families With a Child With and Without Chronic Illness? A Meta-Analysis. *Journal of Pediatric Psychology*, *38*(7), 708–721. https://doi.org/10.1093/jpepsy/jst020

† Pinquart, M. (2014). Associations of General Parenting and Parent–Child Relationship With Pediatric Obesity: A Meta-Analysis. *Journal of Pediatric Psychology*, *39*(4), 381–393. https://doi.org/10.1093/jpepsy/jst144

† Pinquart, M. (2016). Associations of Parenting Styles and Dimensions with Academic Achievement in Children and Adolescents: A Meta-analysis. *Educational Psychology Review*, *28*(3), 475–493. https://doi.org/10.1007/s10648-015-9338-y

* Rodrigues, M., Sokolovic, N., Madigan, S., Luo, Y., Silva, V., Misra, S., & Jenkins, J. (2021). Paternal Sensitivity and Children’s Cognitive and Socioemotional Outcomes: A Meta‐Analytic Review. *Child Development*, *92*(2), 554–577. https://doi.org/10.1111/cdev.13545

† Schulz, S., Nelemans, S., Hadiwijaya, H., Klimstra, T., Crocetti, E., Branje, S., & Meeus, W. (2023). The future is present in the past: A meta‐analysis on the longitudinal associations of parent–adolescent relationships with peer and romantic relationships. *Child Development*, *94*(1), 7–27. https://doi.org/10.1111/cdev.13849

† Valcan, D. S., Davis, H., & Pino-Pasternak, D. (2018). Parental Behaviours Predicting Early Childhood Executive Functions: A Meta-Analysis. *Educational Psychology Review*, *30*(3), 607–649. https://doi.org/10.1007/s10648-017-9411-9

* Zeegers, M. A. J., Colonnesi, C., Stams, G.-J. J. M., & Meins, E. (2017). Mind matters: A meta-analysis on parental mentalization and sensitivity as predictors of infant–parent attachment. *Psychological Bulletin*, *143*(12), 1245–1272. https://doi.org/10.1037/bul0000114
